# Diversity and structure of *PIF/Harbinger*-like elements in the genome of *Medicago truncatula*

**DOI:** 10.1186/1471-2164-8-409

**Published:** 2007-11-09

**Authors:** Dariusz Grzebelus, Slawomir Lasota, Tomasz Gambin, Gregory Kucherov, Anna Gambin

**Affiliations:** 1 Department of Genetics, Plant Breeding and Seed Science, Agricultural University of Krakow, Al. 29 Listopada 54, 31-425 Krakow, Poland; 2 Institute of Informatics, Warsaw University, Banacha 2, 02-097, Poland; 3 Institute of Computer Science, Warsaw University of Technology, Nowowiejska 15/19, 00-665 Warsaw, Poland; 4 LIFL/CNRS/INRIA, Bat. M3 59655 Villeneuve d'Ascq, Lille, France

## Abstract

**Background:**

Transposable elements constitute a significant fraction of plant genomes. The *PIF/Harbinger *superfamily includes DNA transposons (class II elements) carrying terminal inverted repeats and producing a 3 bp target site duplication upon insertion. The presence of an ORF coding for the DDE/DDD transposase, required for transposition, is characteristic for the autonomous *PIF/Harbinger*-like elements. Based on the above features, *PIF/Harbinger*-like elements were identified in several plant genomes and divided into several evolutionary lineages. Availability of a significant portion of *Medicago truncatula *genomic sequence allowed for mining *PIF/Harbinger*-like elements, starting from a single previously described element *MtMaster*.

**Results:**

Twenty two putative autonomous, i.e. carrying an ORF coding for TPase and complete terminal inverted repeats, and 67 non-autonomous *PIF/Harbinger*-like elements were found in the genome of *M. truncatula*. They were divided into five families, *MtPH-A5*, *MtPH-A6*, *MtPH-D*,*MtPH-E*, and *MtPH-M*, corresponding to three previously identified and two new lineages. The largest families, *MtPH-A6 *and *MtPH-M *were further divided into four and three subfamilies, respectively. Non-autonomous elements were usually direct deletion derivatives of the putative autonomous element, however other types of rearrangements, including inversions and nested insertions were also observed. An interesting structural characteristic – the presence of 60 bp tandem repeats – was observed in a group of elements of subfamily *MtPH-A6-4*. Some families could be related to miniature inverted repeat elements (MITEs). The presence of empty *loci *(RESites), paralogous to those flanking the identified transposable elements, both autonomous and non-autonomous, as well as the presence of transposon insertion related size polymorphisms, confirmed that some of the mined elements were capable for transposition.

**Conclusion:**

The population of *PIF/Harbinger*-like elements in the genome of *M. truncatula *is diverse. A detailed intra-family comparison of the elements' structure proved that they proliferated in the genome generally following the model of abortive gap repair. However, the presence of tandem repeats facilitated more pronounced rearrangements of the element internal regions. The insertion polymorphism of the *MtPH *elements and related MITE families in different populations of *M. truncatula*, if further confirmed experimentally, could be used as a source of molecular markers complementary to other marker systems.

## Background

Transposable elements (TEs) are dispersed repetitive sequences constituting a major fraction of plant genomes, ranging from 10% of *Arabidopsis thaliana *genome [1], to an estimated value over 70% of maize genome [2]. Class I elements (retrotransposons), transposing via an RNA intermediate, form the most abundant fraction, while class II elements (DNA transposons), use a 'cut and paste' mechanism for transposition and are usually less numerous.

Advances in genome sequencing of model plant species enabled systematic, computer-based studies towards the identification of repetitive sequences, including those representing putative TEs. The presence of certain structural characteristics of particular groups of TEs allowed the development of a range of strategies for *de novo *or homology-based identification of novel elements. A number of methods for automatic mining of transposable elements were developed [3-6], To date, two model plant genomes, i.e. *A. thaliana *and *Oryza sativa *(rice) have been extensively studied [7-11].

Founder members of the *PIF/Harbinger *superfamily of class II TEs were identified in maize [12] and *A. thaliana *[7]. Other full-length elements were subsequently found in rice (*Pong *[13]), carrot, and *M. truncatula *(*Master *[14]). Autonomous *PIF/Harbinger*-like elements carry 14–25 bp long terminal inverted repeats (TIRs) flanked by 3 bp long (TTA/TAA) target site duplications (TSD), and a DDD/DDE transposase showing similarity to that of the bacterial IS5 insertion sequence. The group of *PIF/Harbinger*-like elements was shown to be widespread in the plant kingdom and composed of two easily distinguishable subgroups, i.e. *PIF *and *Pong *[15]. Elements representing both subgroups were related to certain miniature inverted repeat elements (MITEs), like *Tourist *in maize [12,16] and *mPING *in rice [13].

*Medicago truncatula *(barrel medic) has been chosen as a model plant for the Fabaceae family, primarily to study relationships between plants and their symbiotic microbes. It has a relatively small genome of 500 to 600 Mbp [17], shows annual growth habit and self-fertility. The genome of *M. truncatula *has not been extensively analysed with respect to TE identification. A MITE element *Bigfoot *was reported in the genomes of *M. truncatula *and *M. sativa *[18], a set of *Ty3*/gypsy-like *Ogre *elements characteristic for legume species was described in *M. truncatula *[19], and several other *M. truncatula *elements were briefly characterized in Repbase Update database [20]. A recent study of another model legume, *Lotus japonicus*, identified a number of *PIF*- and *Pong*-like elements and a strong evidence for their recent amplification in the host genome [21].

In this paper we used the accumulated *M. truncatula *genomic sequence data to identify putative TEs belonging to the *PIF/Harbinger *superfamily and related to a previously characterized *MtMaster *element [14]. Therefore, our study was focused on identification and in-depth characterization of a strictly defined group of full-length (putative autonomous and non-autonomous) TEs carrying not only a *PIF/Harbinger*-specific transposase, but also a particular TIR motif characteristic of most of the *PIF*-like, but not of the *Pong*-like elements.

## Results

### Identification and phylogeny of *PIF/Harbinger*-like elements of *M. truncatula*

The initial search of the *M. truncatula *genome aimed at the identification of putative autonomous elements, i.e. those carrying an ORF showing homology to the predicted *MtMaster *TPase (transposase) protein sequence [14] and flanked with terminal inverted repeats of at least 14 bp, containing the G(N)_5_GTT motif, and followed by a 3 bp-long TSD (TAA or TTA). This resulted to 44 sequences showing significant homology (E-value < 10^-20^) to the TPase, after eliminating the redundancy coming from overlapping BACs. We obtained precisely the same hits using the whole TPase sequence and the DDE region, likely because of the very rigorous E-value threshold imposed during the search. Of the identified sequences, 22 were flanked by TIRs and TSDs characteristic for *PIF/Harbinger*-like elements and these were assumed to represent complete transposable elements. They ranged in length from 2,180 to 25,288 bp. In 11 of these elements, another coding region, similar to the *MtMaster *orf1 with E-value ranging from 10^-4 ^to 10^-99^, could be found. The relative order of both ORFs was variable – five elements had orf1 upstream and six downstream the TPase (Table 1).

**Table 1:** Characteristics of the core *PIF/Harbinger*-like elements of *M. truncatula*

Element	GenBank sequence no.	Position (first base-last base)	Element length	TPase/orf1 orientation	No. of introns in TPase
*MtPH-A5-Ia*	AC132565	126754–132718	5965 bp	TP > orf1	2
*MtPH-A6-1-Ia*	AC151598	118204–122278	4075 bp	TP > orf1	2
*MtPH-A6-2-Ia*	AC122722	63283–67500	4218 bp	TP > orf1	2
*MtPH-A6-3-Ia*	AC144563	2339–7183 (-)*	4845 bp	TP > orf1	2
*MtPH-A6-4-Ia*	AC146704	67498–72196	4699 bp	TP > orf1	1
*MtPH-D-Ia*	AC135566	96556–99715 (-)	3160 bp	TP > orf1	1
*MtPH-E-Ia*	AC135606	48232–52188	3957 bp	no orf1	2
*MtPH-E-IIa*	AC139748	47216–50597	3382 bp	no orf1	2
*MtPH-M-1-Ia (MtMaster)*	AC144478	46234–51373	5140 bp	orf1 > TP	1
*MtPH-M-1-IIa*	AC146861	104340–109602 (-)	5006 bp	orf1 > TP	1
*MtPH-M-2-Ia*	AC160098	52670–58188	5519 bp	orf1 > TP	2
*MtPH-M-2-IIa*	AC149306	56522–61824	5303 bp	orf1 > TP	2
*MtPH-M-3-Ia*	CR962122	73712–77759	4048 bp	orf1 > TP	1

* (-) indicates that the sequence of the TE is reverse complement of the original BAC sequence

**Figure 1:**
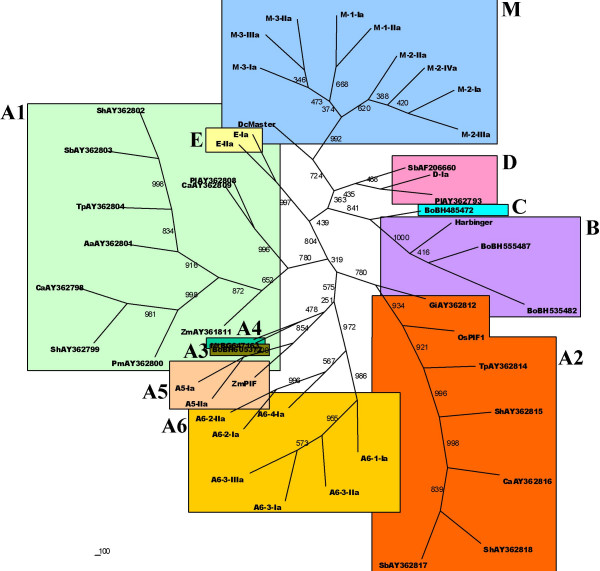
**Neighbor-joining tree representing the diversity of the *M. truncatula PIF/Harbinger*-like elements in relation with other previously identified TEs**. Lineages are marked with color rectangles and letters, numbers show bootstrap values obtained using 1000 replicates.

A phylogenetic analysis of the DDE domain region of the TPase revealed that the *M. truncatula PIF/Harbinger*-like elements could be divided into five lineages. Nine elements, including the previously described *MtMaster*, were grouped into lineage M, together with carrot *DcMaster *[14]. In seven of these elements the orf1 preceded the TPase as expected, while for the remaining two the orf1 was absent, most likely because of an internal deletion. Eight elements formed a new lineage designated as A6. Typically for the group A, the orf1 was located downstream the TPase in the five elements carrying both coding regions. Another new lineage, designated as E, was formed by two elements. In none of them could the orf1 be identified. Two other elements were included into lineage A5, together with maize *ZmPIF *[12] and one was placed into lineage D (Figure 1). However, in the latter case the orf1 was located downstream to TPase, contrary to previously described elements from that lineage [15].

### Diversity and abundance of *PIF/Harbinger*-like elements in *M. truncatula*

In addition to the TPase-containing elements described above, using a strategy outlined in the Methods section, we identified additional 67 elements lacking any coding capacity and thus considered as non-autonomous. List of all identified elements and their coordinates are given in the Additional File 1. The grouping of the identified transposable elements was based on the full element sequence similarity or 5' and 3' terminal sequence similarity using two approaches: hierarchical clustering and multidimensional scaling (Additional File 2). This strategy allowed us to define families and subfamilies of *PIF/Harbinger*-like transposable elements in *M. trunactula *(Table 2), where families essentially reflected the lineages previously identified on the basis of the TPase phylogeny, and subfamilies grouped elements carrying homologous TIRs (Table 3) and showing a degree of overall DNA sequence similarity. For each but two subfamilies, one or two putatively autonomous core elements could be identified. The exception was a low copy number family *MtPH-E*, for which none of the elements contained a region homologous to the orf1.

**Table 2:** Classification and abundance of *M. truncatula PIF/Harbinger*-like elements

Family	Subfamily	Number of elements			
		
		Total	Containing TPase	Containing Tpase and orf1	With no coding capacity
*MtPH-A5*		4	2	2	2
*MtPH-A6*	1	9	1	1	8
	2	6	2	1	4
	3	16	3	2	13
	4	23	2	1	21
*MtPH-D*		1	1	1	0
*MtPH-E*		3	2	0	1
*MtPH-M (MtMaster)*	1	4	2	2	2
	2	5	4	3	1
	3	18	3	1	15

Total:		89	22	14	67

**Table 3:** Consensus TIR sequences of *M. truncatula PIF/Harbinger*-like elements

Family	Subfamily	TIR length	TIR sequence
*MtPH-A5*		21 bp	5' GGGKGYGTTTGTTTGAGGGTT 3'
*MtPH-A6*	1	15 bp	5' GGGTCCGTTTGGTTC 3'
	2	15 bp	5' GGCTMTGTTTGGATT 3'
	3	22 bp	5' GGGTCCGTTTGGTTCGAGARTT 3'
	4	17 bp	5' GGCTTTGTTTGCGAGTT 3'
*MtPH-D*		12 bp	5' GGCTWTGTTTGG 3'
*MtPH-E*		22 bp	5' GGGCCTGTTTGRAACACTTTTT 3'
*MtPH-M (MtMaster)*	1	14 bp	5' GTGYRTGTTTGGYA 3'
	2	14 bp	5' GYRYGTGTTTGGTT 3'
	3	14 bp	5' GNSYSTGTTTGGTT 3'

The largest family, *MtPH-A6*, contained 54 elements, while family *MtPH-D *was represented only by a single element. The second most abundant family, containing 27 elements, was *MtPH-M (Master)*, of which 18 was grouped into subfamily 3.

### Detailed structure analysis of *MtPH *families

*MtPH-A6 *consisted of four subfamilies represented by putative autonomous elements sharing similar ORF organization, i.e. a TPase containing two introns, followed by orf1. *MtPH-A6 *TPases formed a well supported clade, containing four subclades with high bootstrap values, representing the corresponding subfamilies (Figure 1).

Subfamily *MtPH-A6-1 *contained nine elements ranging in length from 802 to 8,707 bp, the longest element carrying a nested insertion of the 7,555 bp long *RAM12 gypsy*-like retrotransposon.

Subfamily *MtPH-A6-2 *grouped six elements, 898 to 4,218 bp long, all being simple internal deletion derivatives of the core element *MtPH-A6-2-Ia*.

**Figure 2:**
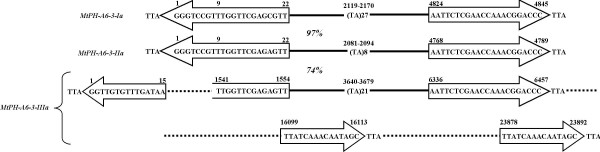
**Structure of three elements representing family *MtPH-A6-3***. Arrows show terminal inverted repeats (TIRs), letters represent sequences of target site duplications (TSDs) and TIRs, solid lines show homologous regions with similarity rate written in italics, dotted lines show regions with no homology, numbers in bold show localization of nucleotide positions of important features, (TA) indicates presence of a microsatellite repeat, followed by the number of the core motif repeats.

Sixteen elements belonged to subfamily *MtPH-A6-3*, ranging in length from 553 to 4,845 bp, except for a much larger, 23,892 bp long element *MtPH-A6-3-IIIa*, initially identified as being flanked by 15 bp TIRs unrelated to those of the *MtPH-A6-3 *subfamily. However, the element contained a 4.9 kb region 74% identical to the two elements mentioned above, but lacking the first 8 bases in the 5' TIR (Figure 2). Hence, the true boundaries of the elements could not be initially identified using our mining strategy. An interesting feature of that subfamily was the presence of a perfect microsatellite site in the first intron of the TPase. The three elements containing the region coding for the TPase, *MtPH-A6-3-Ia*, *MtPH-A6-3-IIa*, and *MtPH-A6-3-IIIa *had, respectively, 27, 8, and 21 repeats of the (TA)_n _core motif (Figure 2).

**Figure 3:**
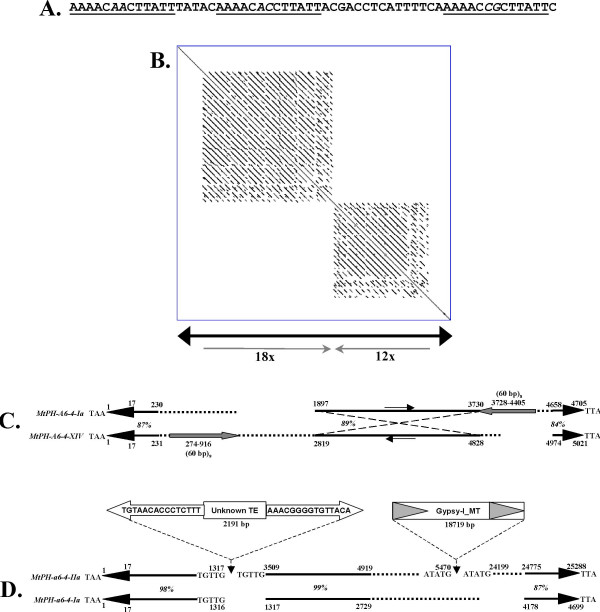
**VNTR regions, inversions, and nested insertions in elements belonging to family *MtPH-A6-4***. A. Consensus sequence of the 60 bp core VNTR motif, triplicated regions within the core motif are underlined, variable nucleotide positions within the triplicated motif are written in italics. B. Dot-plot and schematic representation of *MtPH-A6-4-XXI*, an example of TE carrying a large number of tandem repeats. Thick black arrowheads represent TIRs, gray arrows indicate localization and orientation of the VNTR region, number of repetitions is given below each arrow. C. Comparison of two elements containing an inversion of the internal region, thick black arrowheads show TIRs, gray arrows show localization of the VNTR, thin arrows indicate the orientation of the inverted region, solid lines represent homologous regions with similarity rates written in italics, dotted lines represent regions with no homology, numbers in bold show localization of nucleotide positions of important features. D. Organization of the long element *MtPH-A6-4-IIa *as compared to the core element *MtPH-A6-4-Ia*, thick black arrowheads show TIRs, solid lines represent homologous regions with percentages of similarity written in italics, dotted lines represent regions with no homology, numbers in bold show localization of nucleotide positions of important features, nested TEs are drawn above the *MtPH-A6-4-IIa *element.

*MtPH-A6-4 *subfamily members ranged in length from 431 to 25,288 bp. Among the 23 members of that subfamily, 18 were characterized by the presence of imperfect 60 bp long tandem repeats, variable in number, while in the remaining five elements the core repeat was entirely absent. Each repeat itself contained a triplicated AAACNNCTTATT motif. These elements contained from 2 to 35 repeats that in extreme cases covered almost the entire region between the TIRs (Figure 3A and 3B). In some elements, tandem repeats were present only in one subterminal region, while for the others they were present in both subterminal regions in opposite orientation. The 60 bp tandem repeats were identified in 27 other sites in the *M. truncatula *genome, initially not identified as occupied by *MtPH-A6-4 *elements. However, BLAST search with the terminal 214 bp + 3 bp TSD of the *MtPH-A6-4-Ia *and *MtPH-A6-4-IIa *elements indicated that in all instances at least one of the regions flanking the repeats showed residual homology to the TE terminus (E value < 1e-08). The presence of tandem repeats facilitated internal rearrangements resulting in inversions of the internal region (Figure 3C). Two nested insertions were identified in the longest element *MtPH-A6-4-IIa*, which showed three blocks of significant homology to the *MtPH-A6-4-Ia *core element, interrupted by an unidentified element of 2,191 bp carrying 15 bp TIRs and flanked by a 5 bp long TSD and a *gypsy*-like retrotransposon (Figure 3D).

*MtPH-M *family included three subfamilies with short (14 bp), similar TIRs and orf1 followed by TPase. Subfamily *MtPH-M-1 *contained only four elements, ranging in length from 812 to 5,140 bp. Two of them, *MtPH-M-1-Ia *(previously described as *MtMaster *[10]) and *MtPH-M-1-IIa *(showing 90% overall sequence identity to *MtMaster*) had both ORFs, and the remaining two were internally deleted derivatives.

**Figure 4:**
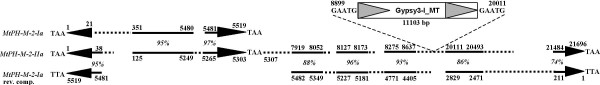
**Mosaic structure of the *MtPH-M-2-IIa *element, as compared to the core element *MtPH-M-2-Ia***. Solid lines represent homologous regions with similarity rates written in italics, dotted lines represent regions with no homology, numbers in bold show the localization of nucleotide positions of important features, a nested retrotransposon is drawn above the AC149306 element.

Five elements were grouped into subfamily *MtPH-M-2*, three of them carrying both orf1 and TPase. The region containing element *MtPH-M-2-IIa *occurred to be a composite structure of two related TEs. The initially identified sequence flanked by TIRs and TSDs spanned over 21,696 bp. The 5,303 bp element *MtPH-M-2-IIa *occupied the 5' region of that sequence, however the downstream sequence also contained blocks of homology to the core element *MtPH-M-2-Ia*, and a nested insertion of a *Gypsy*-like retrotransposon (Figure 4). It indicates that an ancient copy of a TE related to those belonging to the subfamily *MtPH-M-2 *became a target for subsequent nested insertions. Other elements from that family ranged in length from 2,240 to 7,816 bp.

**Figure 5:**
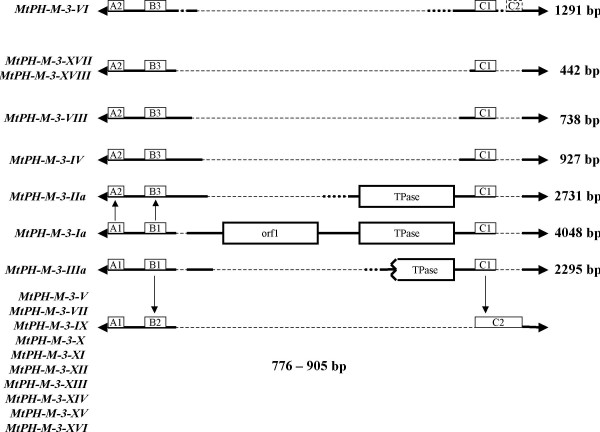
**Intra-family relationships among the *MtPH-M-3 *elements**. Thick solid lines represent homologous regions, thick dotted lines represent regions with no homology, thin dashed lines represent internal deletions, blocks marked with orf1 and TPase show localization of the coding regions, blocks marked with A, B, and C show localization of sequence polymorphisms used to trace intra-family lineages, numbers show the length of the element.

Subfamily *MtPH-M-3 *was the largest within the family and contained 18 elements, of which two carried both ORFs. Their length varied from 442 to 4,048 bp, and interestingly, two 442 bp-long elements were 100% identical. As their length resembled that of miniature inverted repeat elements (MITEs), but unlike MITEs, their number in the *M. truncatula *genome was low, it would be tempting to speculate that these copies might become founders of a new MITE family. A slightly more advanced stage of proliferation of MITE-like elements could be observed with a group of 10 short (776–905 bp) elements from the same family. A more detailed comparison of the element sequences provided a further insight into the evolution of *MtPH-M-3 *subfamily. Internal deletions were accompanied by differentiation and rearrangement of variant sequences (blocks A, B, and C in Figure 5, Additional File 3) in the subterminal regions. Two lineages could be traced that originated from the core element *MtPH-M-3-Ia*, that included respectivley 5 and 11 elements. The element *MtPH-M-3-VI *showed apparently a mosaic structure, as it contained the 3' subterminal region from lineage I, while the major portion of the element contained sequence variants chracteristic for the lineage II (Figure 5).

Family *MtPH-A5 *was represented by four elements ranging in length from 1,182 to 6,770 bp. The two putative autonomous elements were 72% similar over the entire sequence, but within the coding region the nucleotide sequence similarity reached 95%. Two shorter elements were deletion derivatives of full-length elements. Interestingly, a recently reported *MITRAV *family of miniature elements of barrel medic [22] showed a high nucleotide sequence similarity of their termini to the *MtPH-A5 *elements, spanning over ca. 40 bp on both ends of the element.

Family *MtPH-E *consisted of three elements, none of which carried both ORFs. The elements ranged from 1,508 to 3,957 bp. The two largest elements were very similar, differing by one indel, while the similarity of the shortest element to the other two was restricted only to the 180 bp of the 5' terminus and 70 bp of the 3' terminus.

Family *MtPH-D *was represented by a single element of 3,160 bp, carrying both ORFs. However, their orientation was opposite to that of typical *PIF/Harbinger*-like elements representing the D lineage [15]. Its localization in the D lineage was not strongly supported by bootstrap analysis (Figure 1). The fact that no internally truncated elements were identified could suggest that the element might be capable of perfect excision, not triggering the process of abortive gap repair.

### Documentation of the mobility of the mined elements

**Figure 6:**
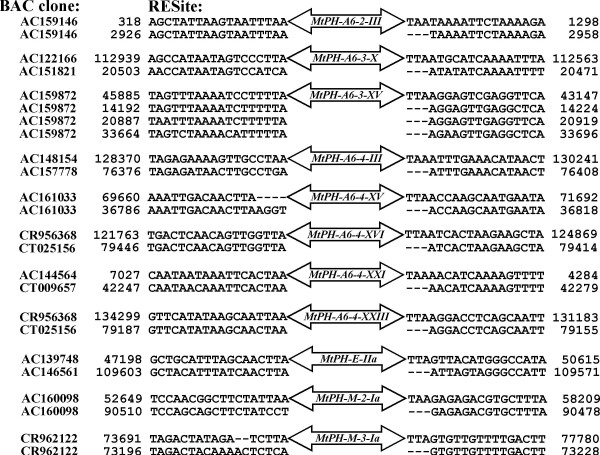
**RESites corresponding to mined *M. truncatula MtPH *elements**. For each group of sequences the upper one represents the insertion site and the lower one is the corresponding RESite. Numbers indicate the nucleotide position of the first and the last nucleotide of the presented sequence, related to the BAC clone from which it was extracted.

In order to find evidence for a possible mobility of identified elements we implemented a strategy proposed by Le et al. [8], i.e. we searched for regions, called RESites (Related to Empty Sites), paralogous to sequences flanking the insertion sites, but lacking the transposable element. We identified 11 RESites, of which five represented insertion sites of non-autonomous elements belonging to the *MtPH-A6-4 *subfamily, while two and one of them were related to non-autonomous elements of the *MtPH-A6-3 *and *MtPH-A6-2 *subfamilies, respectively. The remaining three RESites represented insertion sites of the putative autonomous (core) elements belonging to family *MtPH-E *and subfamilies *MtPH-M-2*, and *MtPH-M-3 *(Figure 6).

We identified several *M. truncatula *ESTs showing high similarity to putative expression products (orf1 and TPase) of the mined autonomous elements (Additional File 4). However, ESTs directly corresponding to the putative expression products, both to the orf1 (CX532696, 641 bp, 94% identity) and the TPase (AW686181, 304 bp, 99% identity), could be detected only in case of elements representing the *MtPH-M-1 *subfamily (Additional File 5). Interestingly, A number of ESTs similar to non-coding terminal regions of the TEs could also be identified (data not presented).

**Figure 7:**
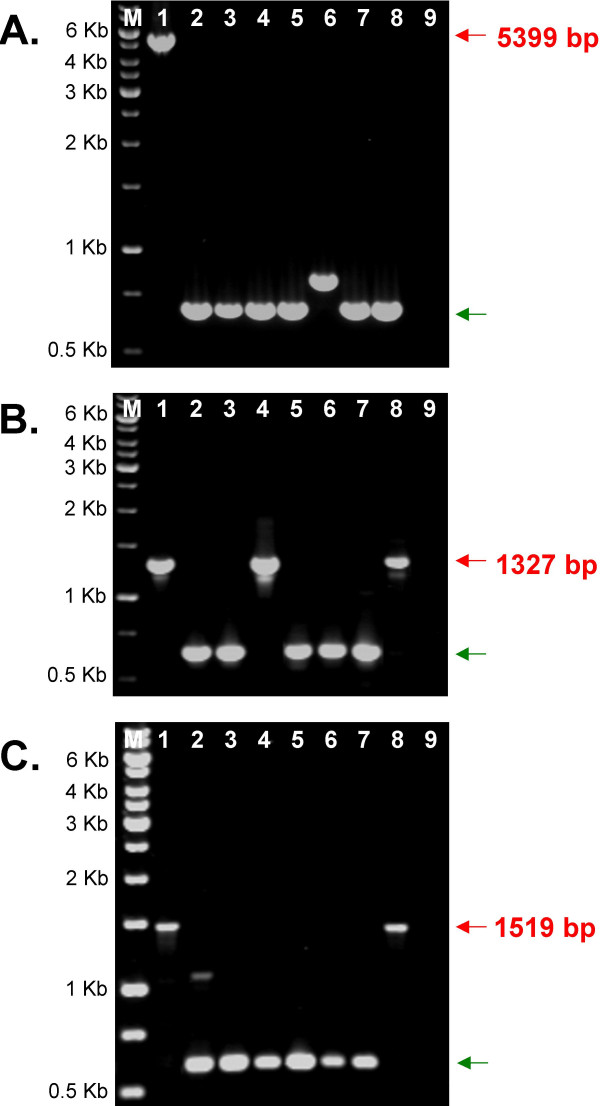
**Insertion related size polymorphisms of MtPH-A6-3 elements**. A. Long PCR amplification of the region encompassing the *MtPH-A6-3-IIa *insertion site, B. PCR amplification of the region encompassing the *MtPH-A6-3-VI *insertion site, C. PCR amplification of the region encompassing the *MtPH-A6-3-XVI *insertion site. Lanes: M – 1 kB ladder (Fermentas), 1 – Jemalong A17, 2 – L163, 3 – L174, 4 – L368, 5 – L530, 6 – L544, 7 – L651, 8 – L734, 9 – negative control. Fragments representing occupied and unoccupied sites are marked by red and green arrows, respectively. Numbers in red indicate the expected length of products representing occupied sites, predicted from the original sequence.

The PCR assay of *MtPH *insertion polymorphism was performed on eight *M. truncatula *populations selected to represent genetic diversity of the species, as proposed by Ronfort [31]. Fifty-six insertion sites identified in the reference genome of cv. Jemalong A17 were checked for presence of the TE. Thirty-seven primer pairs yielded products of the expected size for the reference sample, while 11 generated complex profiles, likely indicating that insertions were present in repetitive regions. The remaining eight primer pairs produced ambiguous results. Of the 37 successful amplifications, 20 occurred to be polymorphic. Usually, the size the shorter amplicon corresponded to the predicted size of the product amplified from the unoccupied site. However, amplicons slightly differing from the expected size were also observed, indicating a possible imperfect excision event (Figure 7).

## Discussion

We developed a strategy for identification of transposable element families through *in silico *genome mining, based on initial assumptions on the type of transposase and the consensus sequences of terminal inverted repeats. It required several consecutive steps, i.e. (1) search for regions coding for the TPase, (2) identification of TIRs flanking the identified regions and matching a defined sequence motif, (3) identification of related elements with no coding capacity, and (4) grouping the identified elements into families on the basis of their sequence similarity. We applied this strategy to mine the genome of *Medicago truncatula *for *PIF/Harbinger*-like elements similar to the previously described *MtMaster *element [14]. In principle, the proposed strategy can be used to mine for any other type of class II TEs, provided that at least one 'seed' element is known.

Diversity of the identified *PIF/Harbinger*-like elements is high, although our search was limited by a specifically defined core TIR sequence. We focused on 22 ORFs coding for putative TPases, representing a half of all initially identified ORFs, as for the other half, TIRs flanking the ORF and containing the required motif could not be found. A recent broad analysis of the TE landscape in another legume, *Lotus japonicus *[21], revealed a presence of nine putative autonomous *PIF*-like elements (besides several more distantly related *Pong*-like elements) in ca. 32 Mb portion of the genome. This number is in agrrement with our results, as we found 22 full-length elements (2.5 times more) in ca. 200 Mb representing a certain level of redundancy. Interestingly, all *PIF*-like TEs from *L. japonicus *represented the A3 lineage, while no A3 members were identified in *M. truncatula*, which may indicate a strikingly different evolutionary fate of that group of TEs in each of the closely related species.

Detailed structure analysis of the mined element families indicates that their proliferation in the genome generally follows the model of abortive gap repair (AGR), as proposed for the *Ac/Ds *elements in maize [23]. Members of a particular family were usually direct deletion derivatives of the related, putative autonomous element. However, assuming that members of all *PIF/Harbinger*-like TE families in the genome of *M. truncatula *were mobilized with similar frequency, the efficiency of AGR seems to vary from one family to another. Two families, *MtPH-A6 *and *MtPH-M*, were the most numerous, while the remaining three were represented by a very small number of copies. Difference in the copy number may be a result of different transposition rates, but it may also indicate that some elements less efficiently trigger the process of AGR following excision, which would result in a higher frequency of perfect excision. The latter is further supported by two observations. Firstly, the members of subfamily *MtPH-A6-4 *contain a variable number of 60 bp tandem repeats in one or both subterminal regions, serving as targets for AGR and leading to increase of the TE copy number accompanied by changes in the number of VNTRs. The presence of 60 bp tandem repeats was inherently connected with *MtPH-A6-4 *elements throughout the *M. truncatula *genome, which implies that they likely evolved in the course of the proliferation of that subfamily. Probably, triggering the AGR from the VNTR region also led to an inversion of the internal region in *MtPH-A6-4-XIV*, as compared to *MtPH-A6-4-Ia*. Secondly, at least one member of the low copy number family *MtPH-E *was transpositionally active, as confirmed by the presence of the RESite, but despite the potential for mobility, the number of *MtPH-E *elements has remained low.

*PIF/Harbinger*-like elements are ancestors of certain groups of miniature transposons (MITEs), the relation of maize *PIF *element and MITEs belonging to the *Tourist *family has been well documented [12,16]. Also, several other MITE families, e.g. *Heartbreaker *from maize [24], *Kiddo *from rice [25], and *Krak *from carrot [14] show TIR sequence similarities to those of *PIF/Harbinger*-like elements. We were able to directly link the previously identified *MITRAV *MITE family [22] to family *MtPH-A5 *of *M. truncatula PIF/Harbinger*-like elements. This suggests that both *MtPH-A5 *and *MITRAV *originated from a recent common ancestor and *MtPH-A5 *TPase might be the *trans*-acting factor for *MITRAV *mobilization, as experimentally proven for the *Pong *and *mPing *MITE in rice [13,26,27]. Also, two groups of two and ten TEs, all classified in the subfamily *MtPH-M-3*, might represent newly emerging MITE families. We performed an initial search for other MITEs showing a TIR homology to the consensus motif of the *PIF/Harbinger *TIRs leading to an identification of few other MITE families (data not presented). Altogether, it confirms that *PIF/Harbinger*-like elements and related MITEs are present in the genome of *M. truncatula*, similar to genomes of other plant species. However, the number of MITE copies is probably much lower than that present in the grass genomes.

A more detailed experimental evaluation of *MtPH *TEs diversity in a range of *M. truncatula *populations should be useful to further characterize the transpositional activity and the dynamics of particular families. Analysis of RESites and a high incidence of insertion related size polymorphisms shows that a significant fraction of the mined elements was mobile in the recent past. The presence of ESTs related to ORFs of the *MtPH *elements, including those directly derived from the *MtPH-M-1 *elements, suggests that they can still be mobile. As proven previously, one transcriptionally active autonomous element can cause *trans*-mobilization of a range of related, but not directly derived elements [13].

Polymorphic insertion sites could be used as a source of molecular markers, as shown previously for other species [28-30], to measure intraspecific diversity in relation to its geographic structure, complementing other molecular marker systems, e.g. these based on microsatellites [31].

## Conclusion

Starting from a single previously described *PIF/Harbinger*-like TE of *M. truncatula*, we identified 89 elements representing the diversity of this superfamily in the host plant genome. They were divided into five families representing different evolutionary lineages, and further into subfamilies. Elements within each subfamily evolved essentially following the model of AGR, leading to the reconstruction of an internally deleted copy in the donor site following transposition. It is likely that different families vary in their potential to trigger the process of AGR. One peculiarity observed in a group of elements representing subfamily *MtPH-A6-4 *was the presence of 60 bp long VNTRs in one or both subterminal regions or even spanning over the entire internal region of the TE. Some of the identified elements are closely related to several MITE families, including a previously described *MITRAV *family. Also, some of the newly identified short elements can be viewed as *in statu nascendi *MITEs, provided that conditions for a rapid burst of their mobility would be met. Further investigation is necessary for a more detailed evaluation of the copy number, transpositional activity, and insertional polymorphism of the TEs, including MITEs, as they could be utilized as a source of molecular markers.

## Methods

### Semi-automated mining of *PIF/Harbinger*-like elements

The experiment was performed on the *M. truncatula *genomic DNA sequence database consisting of 1540 BACs, updated Aug 2005 [32]. As the size of the whole *M. truncatula *genome ranges from 500 to 600 Mbp [17] and the average non-overlapping coverage by each BAC was ca. 100 Kb [32], we estimated that the input sequence data amounted 26–30% of whole genome.

The predicted protein sequence of DDE domain and the whole TPase sequence of the previously identified *MtMaster *element [14] was used as the initial query for a TBLASTN search against the BAC sequence database, using the E-value threshold of 1e-20. The output file was then processed to eliminate redundancy coming from overlapping BACs, and significant hits were extracted, along with up to 30 kb flanking sequences. The extracted sequences were scanned for the presence TIRs and TSDs, using a newly developed tool named TIRfinder, identifying TIRs and TSDs and returning a file with a list of found elements fulfilling user-defined requirements. To provide fast computation on whole genome, the algorithm uses very efficient data structures, such as suffix trees. TIRfinder is an open source software accessible online [33]. The program was written in Java and can be run on Windows or Linux.

We allowed up to four mismatches inside 14 bp of the TIRs and no mismatch in TSDs. Another condition was the presence of the conserved G(N)_5_GTT motif at the 5' end of the TIR. *In silico *prediction of the presence of coding regions was performed for all identified sequences using FGENESH [34].

To identify internally deleted copies of elements related to those found previously, 217 bp-long (3 bp TSD + 14 bp TIR + 200 bp subterminal sequence) terminal regions were extracted from all putative autonomous elements. These sequences were used to scan the *M. truncatula *genomic DNA sequence database (BLASTN, E-value threshold – 1e-10), and regions showing homology to any of the terminal regions were identified. The output was automatically filtered to find sequences of length ranging from 400 to 30,000 bp, flanked with TIRs showing homology to the same autonomous element on both ends. All newly found sequences have been checked whether they contained a region coding for the TPase. All TEs were scanned using Censor [35], to identify the presence of nested elements.

### Phylogenetic analyses, grouping, and visualization of TE sequence similarity

Multiple alignment of 48 transposase sequences of *PIF/Harbinger*-like transposable elements was obtained using T-Coffee [36]. Bootstrap analysis was performed with PHYLIP using seqboot, neighbor, protdist and consense programs [37]. The sequence similarity of 89 TEs was analyzed by the hierarchical clustering method and visualized with help of multidimensional scaling. For both tasks we used the R statistical environment [38]. As a measure of dissimilarity between sequences we used the E-value of BLAST. Hierarchical cluster analysis of a set of dissimilarities was done by hclust (complete linkage) method [39]. Multidimensional scaling [40] visualization is primarily dependent on the analogy of similarity and proximity (and hence of dissimilarity and distance). It re-scales a set of dissimilarity data into distances and produces the low-dimensional configuration that generated them. The visualization for our data was obtained with isoMDS R procedure.

### TE structure analysis

Sequences were visually compared, aligned, edited, and analysed using BioEdit and the included accessory applications [41]. Pairwise sequence comparisons were performed using 'blast 2 sequences' [42] and Yass [43,44]. Dot-plots were generated using Nucleic Acid Dot Plots [45] with a window size of 25 nucleotides and a mismatch limit of 5 positions. Tandem repeats identification was performed using 'mreps' software [46,47].

### Documentation of mobility

In order to find RESites (Related to Empty Sites) in the *M. truncatula *genome we performed a computer-based search, essentially as described by Le et al. [8]. Briefly, we extracted 1 Kb sequence flanking both sides of each of the mined elements, combined them into one sequence of 2 Kb, and used it as a query for a BLASTN search on the whole BAC sequence database. Hits spanning on both sides of the insertion were considered as those representing RESites.

EST search was performed using nucleotide sequences of the putative autonomous elements, using a BLAST tool run against the *M. truncatula *EST database [48].

### PCR conditions

PCR assay was performed on plants representing cv. Jemalong A17 and seven populations from the core *M. truncatula *collection (CC8, as described by Ronfort et al. [31]). Primer pairs were anchored in the regions flanking the mined elements. They were designed using Primer3 [49] to obtain amplification of ca. 600 bp long fragment for the putative empty site. Two cycling protocols were employed. For TEs of length not exceeding 2 Kb a standard PCR was performed. The reaction was set up in the volume of 20 μl and contained 0.25 mM each dNTP, 2 mM MgCl_2_, 10 pmol of each primer, 1 unit of TAQ polymerase (Fermentas) and 2 μl of the PCR buffer supplied by the manufacturer. The thermal profile of the reaction was as followed: 94°C for 2 min., 35 cycles of: 94°C for 30 s, 53°C for 30 s, and 68°C for 90 s, and completed with 68°C for 5 min. For larger elements we used long PCR protocol. Amplification was performed in the volume of 20 μl containing 0.25 mM each dNTP, 10 pmol of each primer, 0,5 unit of long PCR enzyme mix (Fermentas) and 2 μl of the Long PCR buffer supplemented with MgCl_2 _(Fermentas), using the following thermal profile: 94°C for 2 min., 35 cycles of: 94°C for 15 s, 53°C for 30 s, and 68°C for 7 min., and completed with 68°C for 10 min. All reactions were carried out in the Mastercycler or Mastercycler Gradient (Eppendorf). Amplification products were separated on 1% agarose gels and visualized with ethidium bromide under UV.

## Authors' contributions

DG developed strategy for the study, performed the fine-scale analysis of the TEs, performed the PCR, and prepared the final version of the manuscript, SL and TG developed algorithms for TE identification, TG edited HC and MDS graphs, GK analysed tandem repeats, and AG participated in the design of the study, performed HC and MDS analyses, and participated in drafting the manuscript. All authors read and approved the final manuscript.

## Supplementary Material

Additional file 1 List of all *PIF/Harbinger*-like elements identified in the course of the study in the genome of *Medicago truncatula*. Click here for file

Additional file 2 **Similarity-based grouping of *M. truncatula PIF/Harbinger*-like elements.**. Results of multidimensional scaling (MDS): A. whole TE sequence, B. 5'end subterminal regions, C. 3'end subterminal regions, and hierarchical clustering (HC): D. whole TE sequence, E. 5'end subterminal regions, F. 3'end subterminal regions. Click here for file

Additional file 3 Sequence alignment of the A and B blocks differentiating individual elements belonging to the *MtPH-M-3 *family. Click here for file

Additional file 4 **Identification of *M. truncatula *ESTs similar to putative expression products of orf1 and TPases coded by MtPH elements**. Sequence of the whole element was used as query against *M. truncatula *EST database, hits in orf1 and TPase coding regions with E value lower than 1e-06 were scored. Nearly identical hits to orf1 and TPase of the *MtPH-M-1 *elements are marked red. Click here for file

Additional file 5 **Alignment of *MtPH-M-1-Ia *and the ESTs corresponding to orf1 (CX532696) and TPase (AW686181)**. Predicted exons of the orf1 and TPase are highlighted yellow and green, respectively, TSDs of the element are marked gray. Click here for file
