# Supplementary figures and images for: Diversity and structure of PIF/Harbinger-like elements in the genome of Medicago truncatula
Source: BMC Genomics. 2007 Nov 9;8:409. doi: 10.1186/1471-2164-8-409 (PMC2213677; doi:10.1186/1471-2164-8-409)

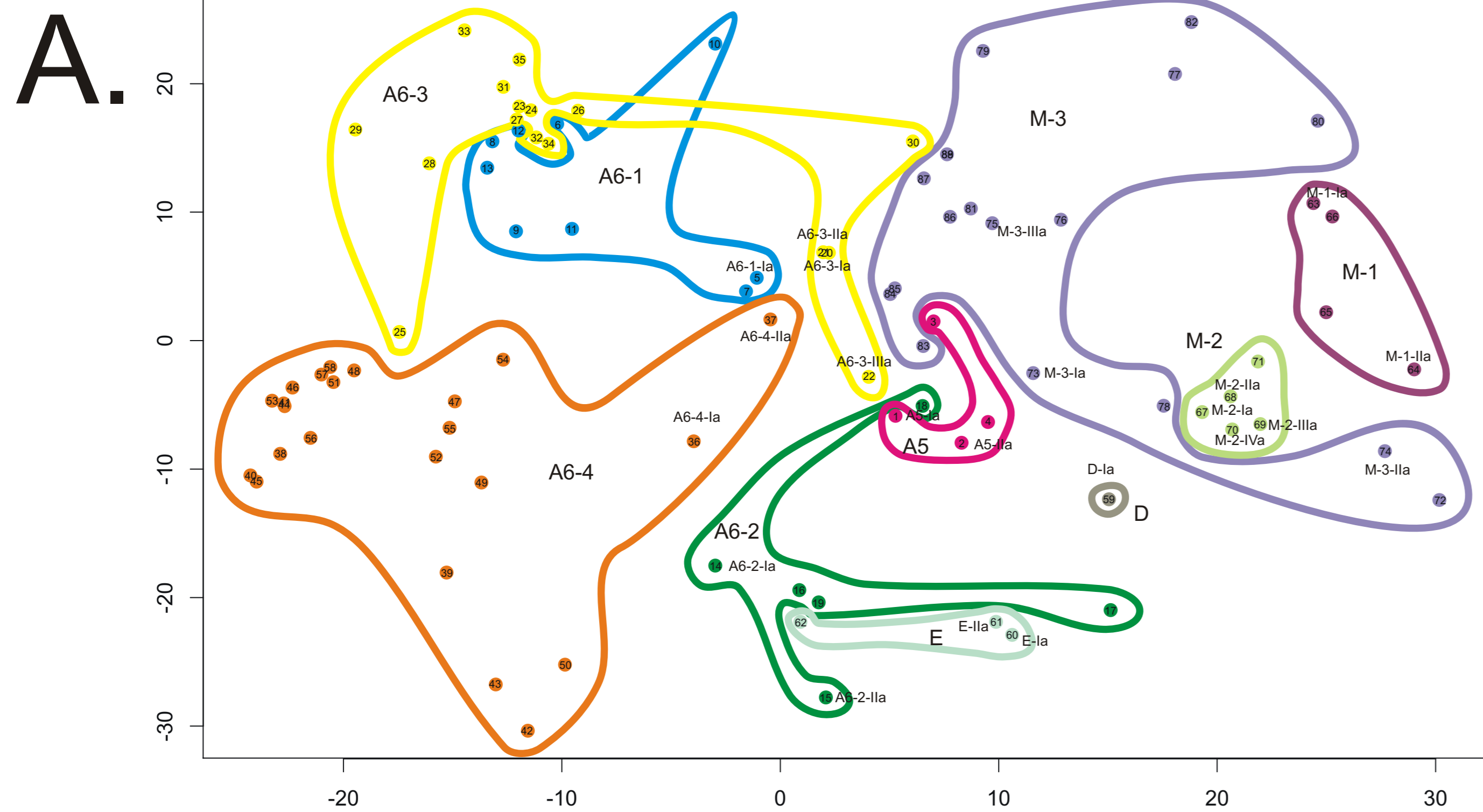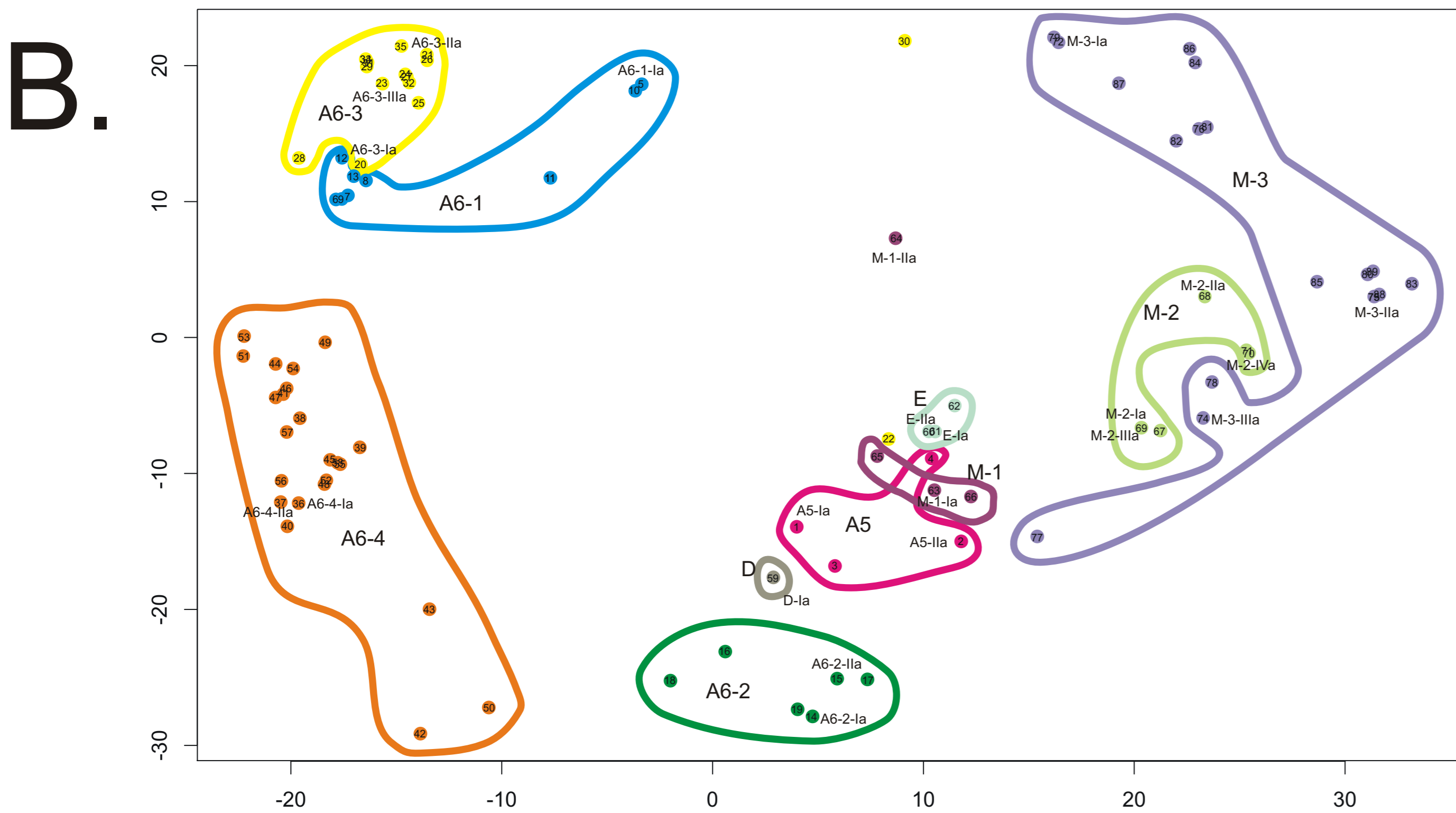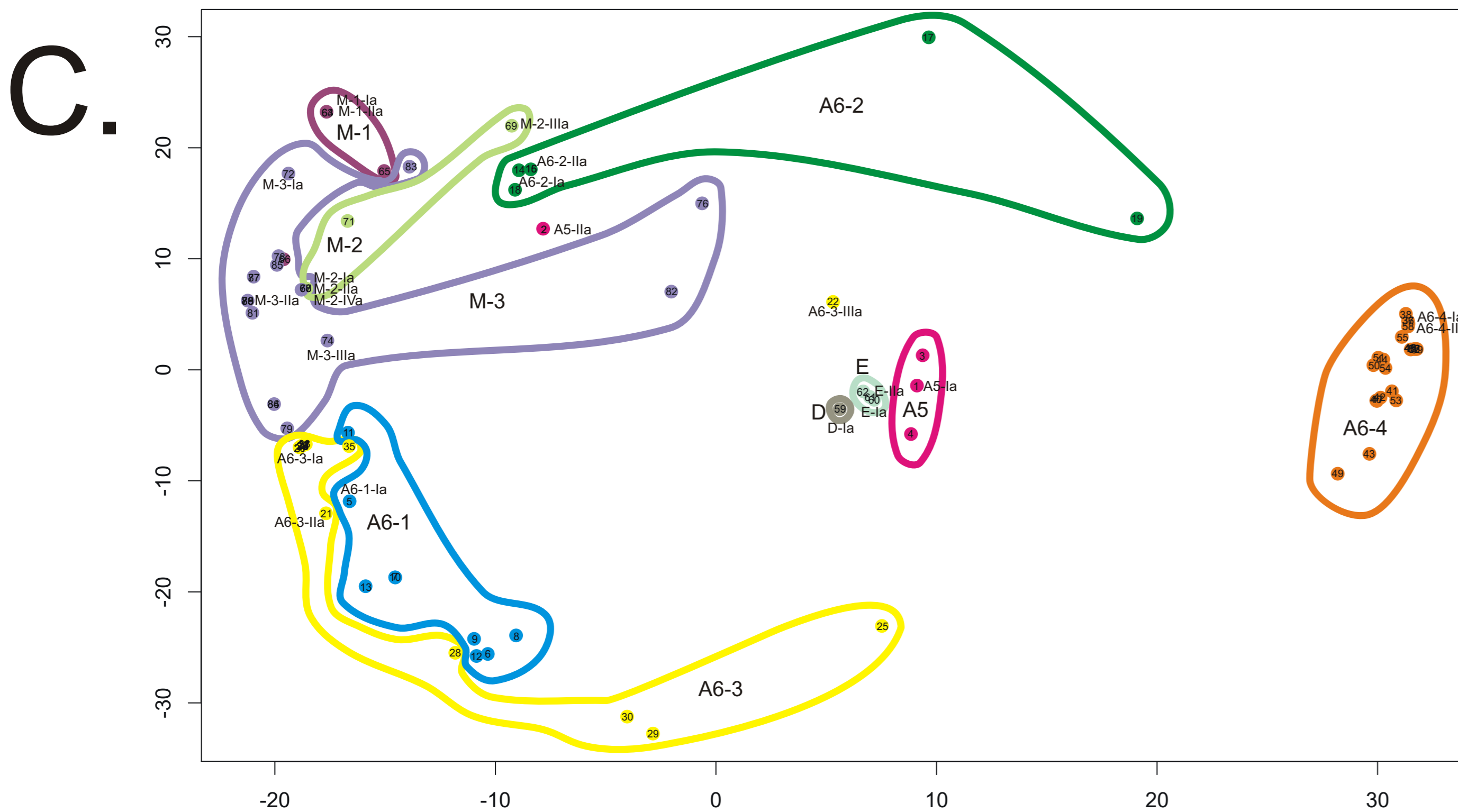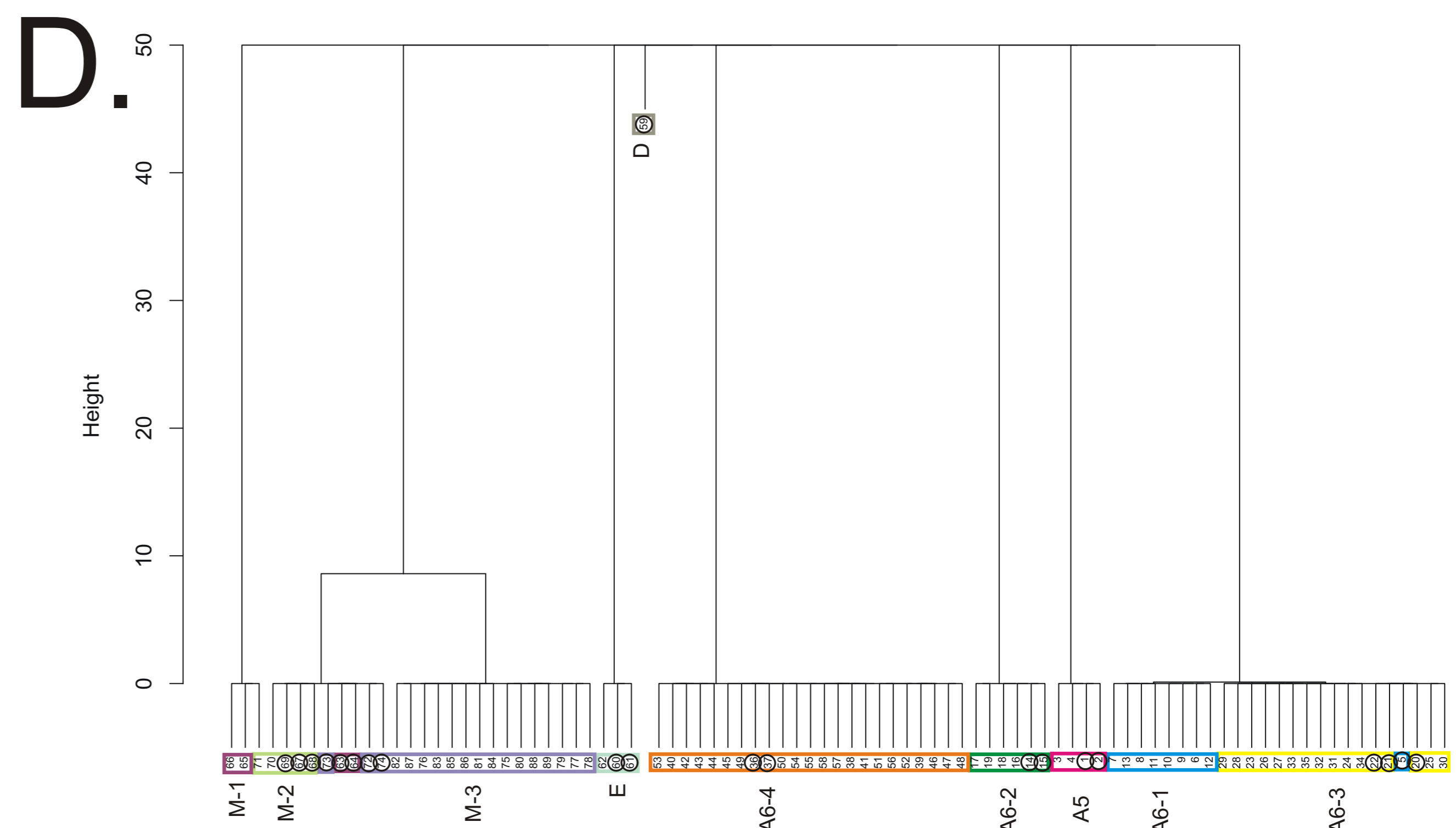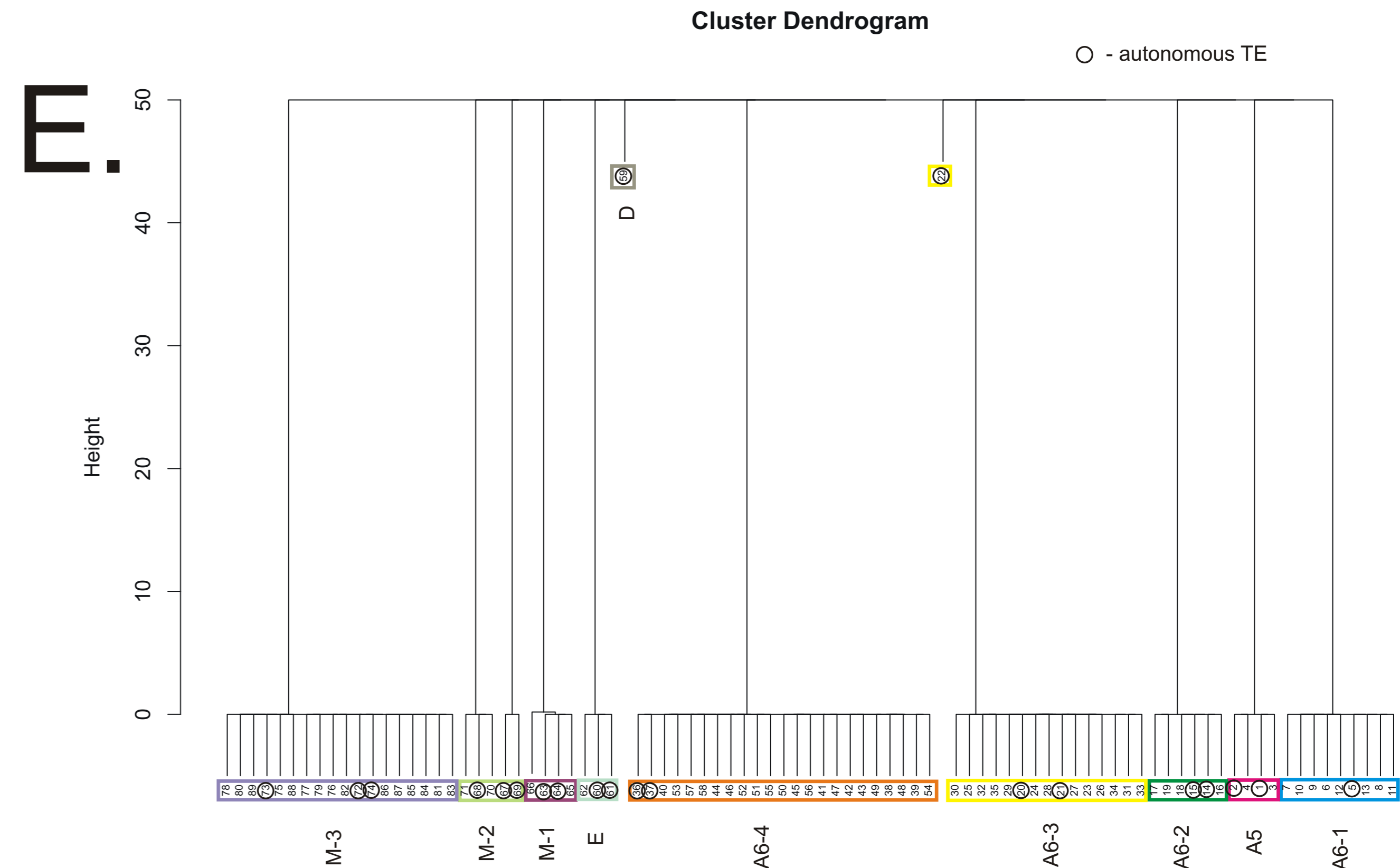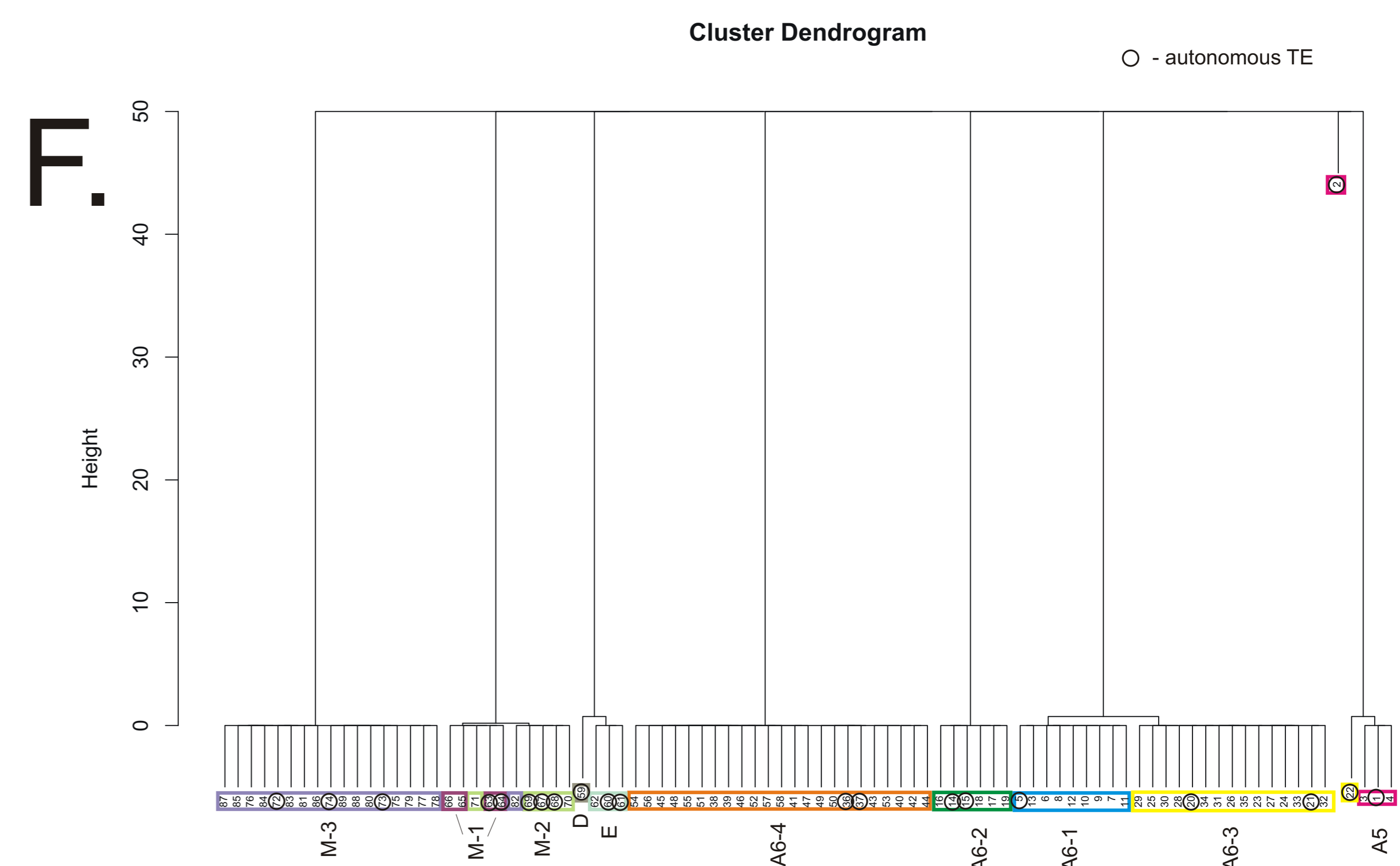

Supplement: Additional file 2 — Similarity-based grouping of M. truncatula PIF/Harbinger-like elements.. Results of multidimensional scaling (MDS): A. whole TE sequence, B. 5'end subterminal regions, C. 3'end subterminal regions, and hierarchical clustering (HC): D. whole TE sequence, E. 5'end subterminal regions, F. 3'end subterminal regions. [file 1471-2164-8-409-S2.pdf]

## Slide 1
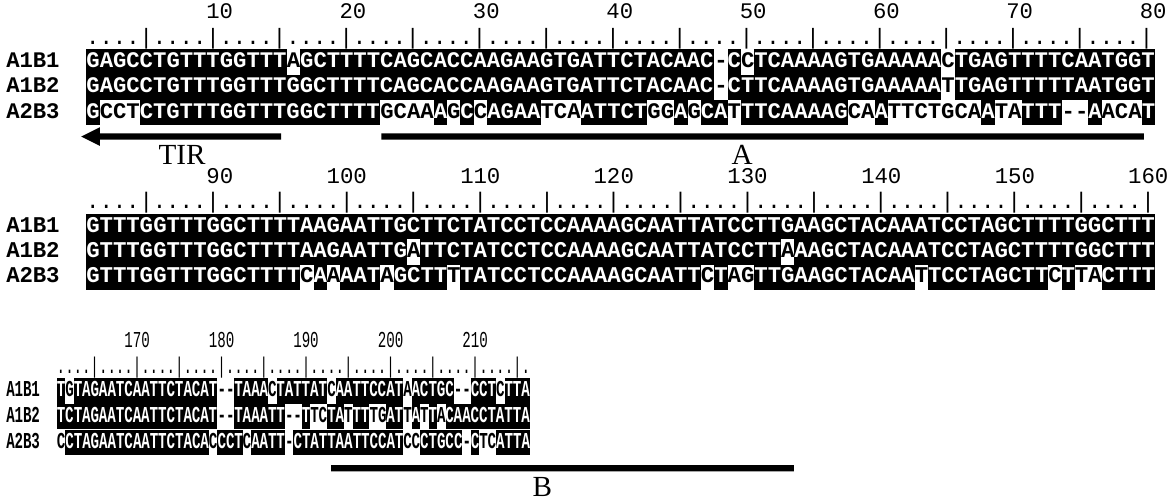

TIR
A
B

Supplement: Additional file 3 — Sequence alignment of the A and B blocks differentiating individual elements belonging to the MtPH-M-3 family. [file 1471-2164-8-409-S3.ppt]
