# Supplementary material for: Diversity and structure of PIF/Harbinger-like elements in the genome of Medicago truncatula
Source: BMC Genomics. 2007 Nov 9;8:409. doi: 10.1186/1471-2164-8-409 (PMC2213677; doi:10.1186/1471-2164-8-409)
Supplement: Additional file 4 — Identification of M. truncatula ESTs similar to putative expression products of orf1 and TPases coded by MtPH elements. Sequence of the whole element was used as query against M. truncatula EST database, hits in orf1 and TPase coding regions with E value lower than 1e-06 were scored. Nearly identical hits to orf1 and TPase of the MtPH-M-1 elements are marked red. [file 1471-2164-8-409-S4.pdf]

Element	EST/orf1		EST/TPase	
	EST code	E value	EST code	E value
MtPH-A5-Ia	TC98869	2.2e-45	BG647163	2.9e-06
MtPH-A5-IIa	-	-	AW686181	7.6e-06
MtPH-A6-1-Ia	-	-	BG647163	5.6e-07
MtPH-A6-2-Ia	-	-	BG647163	3.6e-08
MtPH-A6-2-IIa	-	-	BG647163	5.3e-09
MtPH-A6-3-Ia	-	-	TC111585	6.4e-24
MtPH-A6-3-IIa	-	-	BG647163	1.3e-10
MtPH-A6-3-IIIa	-	-	TC111585	1.6e-23
MtPH-A6-4-Ia	-	-	BM814961<br>BG647163	4.7e-23<br>2.4e-20
MtPH-A6-4-IIa	-	-	-	-
MtPH-D-Ia	AL374958	1.7e-16	AL374957<br>BE317368<br>TC95526	1.8e-61<br>2.0e-38<br>6.5e-16
MtPH-E-Ia	-	-	BI271381<br>TC95526<br>BG647163	4.2e-70<br>2.5e-17<br>2.3e-13
MtPH-E-IIa	-	-	BI271381<br>TC95526<br>BG647163	2.2e-71<br>2.2e-12<br>2.2e-07
MtPH-M-1-Ia	CX532696	1.2e-126	AW686181<br>TC95526<br>BG647163	2.0e-61<br>7.8e-27<br>7.8e-14
MtPH-M-1-IIa	CX532696	2.6e-126	AW686181<br>TC95526<br>BG647163	1.7e-61<br>1.4e-36<br>7.2e-14
MtPH-M-2-Ia	CX532696	1.1e-40	AW686181<br>BG647163<br>TC95526	3.9e-36<br>4.4e-14<br>7.6e-06
MtPH-M-2-IIa	CX532696	1.5e-61	AW686181<br>TC95526	2.2e-37<br>3.0e-07
MtPH-M-2-IIIa	-	-	AW686181<br>BG647163	7.2e-38<br>5.2e-19
MtPH-M-2-IVa	-	-	AW686181	4.3e-29
MtPH-M-3-Ia	CX532696	3.5e-57	AW686181<br>TC95526<br>BG647163	2.8e-43<br>5.0e-28<br>3.4e-12
MtPH-M-3-IIa	-	-	AW686181<br>TC95526<br>BG647163	1.6e-44<br>1.5e-27<br>3.3e-11
MtPH-M-3-IIIa	-	-	TC95526	2.7e-26
