# Supplementary material for: Diversity and structure of PIF/Harbinger-like elements in the genome of Medicago truncatula
Source: BMC Genomics. 2007 Nov 9;8:409. doi: 10.1186/1471-2164-8-409 (PMC2213677; doi:10.1186/1471-2164-8-409)
Supplement: Additional file 5 — Alignment of MtPH-M-1-Ia and the ESTs corresponding to orf1 (CX532696) and TPase (AW686181). Predicted exons of the orf1 and TPase are highlighted yellow and green, respectively, TSDs of the element are marked gray. [file 1471-2164-8-409-S5.pdf]

```
                  10        20        30        40        50        60        70        80        90       100       110       120
          ....|....|....|....|....|....|....|....|....|....|....|....|....|....|....|....|....|....|....|....|....|....|....|....|
AC144478  TTAGTGCATGTTTGGTAAAGCTTCTTCACGCCTCAAACCCACGTTTTCTTCTCCAAACGCGAGTTCGGCGGTTCCTATGATGTTTGGATGAATTCAAACGCGATTCTGTAGATTGTACGG

                 130       140       150       160       170       180       190       200       210       220       230       240
          ....|....|....|....|....|....|....|....|....|....|....|....|....|....|....|....|....|....|....|....|....|....|....|....|
AC144478  CAAGTGCTCAAACGCGGTTTGGGATGACGCTACAAATCTGAGCTTCTGCGGCAGATCAAAATCACTTTTCAACCTTTAATTTCTCAAGGTACAAGTACTTACCTAATTACCCCTAAATTC

                 250       260       270       280       290       300       310       320       330       340       350       360
          ....|....|....|....|....|....|....|....|....|....|....|....|....|....|....|....|....|....|....|....|....|....|....|....|
AC144478  AAGCCTTCAATTTTTCTTTCTCCTAGTTTTCTTCCTTTTCATTACTTCACATCACTTCAACCTTAAATTTTCTGCTACTTGGGAAGATTTTGCAAATCCAAGCTTTCAATTTTCTTCAGA

                 370       380       390       400       410       420       430       440       450       460       470       480
          ....|....|....|....|....|....|....|....|....|....|....|....|....|....|....|....|....|....|....|....|....|....|....|....|
AC144478  TTTTCCTCTATTTTTTCAGATTTTGTGGTTAATCTCATTACTCATGGTAGATGTGTTGGTTGGGTGAATTTATTCATTCATCTTTCCCCTATATATGATGCTTATGAGCTACTTGTGAGA

                 490       500       510       520       530       540       550       560       570       580       590       600
          ....|....|....|....|....|....|....|....|....|....|....|....|....|....|....|....|....|....|....|....|....|....|....|....|
AC144478  ATCATTTCATTGTTCTGCCAAGGCCTAAAGGTAAATACATATTCATAAAAAAAAATTGCTTTGTTTTCTTGCTTCAAAACTGTTAATTTCTATCTCTATTTGCTCTAGTTGTTATAAATT

                 610       620       630       640       650       660       670       680       690       700       710       720
          ....|....|....|....|....|....|....|....|....|....|....|....|....|....|....|....|....|....|....|....|....|....|....|....|
AC144478  TTACCTTAATTTTTTCATCCTGTATCAATGCATTGTATGTGTTTGATAAATAGTCTCAAAGAAGAAAACAATTTTCTTATTGATATGTTTATGATGACTGTTCTATTATTATTGACATGT

                 730       740       750       760       770       780       790       800       810       820       830       840
          ....|....|....|....|....|....|....|....|....|....|....|....|....|....|....|....|....|....|....|....|....|....|....|....|
AC144478  TTATGTTTTTTTTTCTCTCCTGTTGTTCTGTTTTGAGCAATGAGTGTATTGTTTTTGTTTAAAGTTACTTGGTGTGAGTTTAAATTAATTGAACTTGAATGAAATAATGTAAGAAAAGCA

                 850       860       870       880       890       900       910       920       930       940       950       960
          ....|....|....|....|....|....|....|....|....|....|....|....|....|....|....|....|....|....|....|....|....|....|....|....|
AC144478  ACTTACAACTTCAAGCCATTGTTGTATTACTAATTTTTATGTATTTTCATTGTTAGATAAACCTTTTCCAACCTTTACAAAAAAGAGTTACATTTAGCACAAAGTAACAGAACTTCTTTT

                 970       980       990      1000      1010      1020      1030      1040      1050      1060      1070      1080
          ....|....|....|....|....|....|....|....|....|....|....|....|....|....|....|....|....|....|....|....|....|....|....|....|
AC144478  AGTATTTTTGTTTTGTTTTCTGCTATCATTTTCATATGCACAAAACAAGGATATTGGTGTCAGTTTGATCAAGTTCCTTATGTATAGTTTGTTTCATTTTTTTATCATACTTTTGTGTAT

                1090      1100      1110      1120      1130      1140      1150      1160      1170      1180      1190      1200
          ....|....|....|....|....|....|....|....|....|....|....|....|....|....|....|....|....|....|....|....|....|....|....|....|
AC144478  ATAGTATTATGGACTCACTTAAGAGGAAAGTTTCTGCTAACCCCCCATCAACTAACAATGAGGGTCAAAGTTCCAAAGCTAGTTGGAGGGATATTAAAGCCACTGAGTACTTTGTGAAGG

                1210      1220      1230      1240      1250      1260      1270      1280      1290      1300      1310      1320
          ....|....|....|....|....|....|....|....|....|....|....|....|....|....|....|....|....|....|....|....|....|....|....|....|
AC144478  CATGTTTGGATCAAGTTACCAAGGGTCAACGAAATGGTACTTGTTTTACCAAGAAAGGATGGCAAGGTATTGTTTCCCAATTTCATGAACAAAGTGGACTGAATTATGACAAGGTACAAT

                1330      1340      1350      1360      1370      1380      1390      1400      1410      1420      1430      1440
          ....|....|....|....|....|....|....|....|....|....|....|....|....|....|....|....|....|....|....|....|....|....|....|....|
AC144478  TGAAGAATAGGTATGATAGCTTGAGAAAGGAATGGAAAGTATGGTATAACTTGTTTGGAAAAGTTACAGGATTAGGATGGAATTTTGAGAAGAACACCGTTGATGCATCCGATGAGTGGT
                     CX532696 ->      CACGAGGAATGTATGGTATAACTTGTTTGGAAAAGTTACCGGATTAGGATGGAATTTTGAGAAGAACACCGTTGATGCATCTGATGAGTGGT

                1450      1460      1470      1480      1490      1500      1510      1520      1530      1540      1550      1560
          ....|....|....|....|....|....|....|....|....|....|....|....|....|....|....|....|....|....|....|....|....|....|....|....|
AC144478  GGGAGAAGAAAGAATTGGTATGTGAATCACAAATATTTTGTTTAGGATACTTTTATGTTTATATTTACCATAGTTTTATATGTTAGGTTATGAATATGCAGGAAAATCCTCAATATGCAA
          GGGAGAAGAAAGAATTG------------------------------------------------------------------------------------GAAAATCCTCAATATGCAA

                1570      1580      1590      1600      1610      1620      1630      1640      1650      1660      1670      1680
          ....|....|....|....|....|....|....|....|....|....|....|....|....|....|....|....|....|....|....|....|....|....|....|....|
AC144478  AGTTTAGAGACAAGGGACTTCCATTTGCTCACCAACTAACCACACTTTTCAAGGATGTAGTGGCTAATGGAGAGCATGCTTGGGCACCATCAAGTGGTGTATTACCTAATGAGAACTTGG
          AGTTTAGAGACAAGGGACTTCCACTTGCTCACCAACTAACCACACTTTTCAAGGATGTAGTGGATAATGGAGAGCATGATTAGGCACCATAAAGTGGTGTATTACCTAATGAGAACTTGG

                1690      1700      1710      1720      1730      1740      1750      1760      1770      1780      1790      1800
          ....|....|....|....|....|....|....|....|....|....|....|....|....|....|....|....|....|....|....|....|....|....|....|....|
AC144478  GTAATGATGATATTGATGTTGGCTTGGATGATGCAGAAGGTTCGGGTGATAGTGAAGATGCAAGCATTGGAGCAGCAACTGGTTTTGGAAATATTAACTTGAATACATCACAAGGAGCTG
          GTAATGATGATATTGATGTTGGCTTGGATGATGCAGAAGGTTCGGGTGATAGTTAAGATGAAAGCATTGGAGCAACAACTAGTTT-GGAAATATAAACTTGAATACATCACAAGGAGCTG

                1810      1820      1830      1840      1850      1860      1870      1880      1890      1900      1910      1920
          ....|....|....|....|....|....|....|....|....|....|....|....|....|....|....|....|....|....|....|....|....|....|....|....|
AC144478  TTAGTCAAAGTAGTGGACAAAAGAGAAAGAGAGTTATTGGGGCTGAACAGAAAGGAAAGAAAAAAGCTACTCCTTCAACGTCAATAGCTGAGGCTGTTAATGTTATTGCGGAGACTTGCA
          TTAGTCAAAGTAGTGGGCAAAAGAGAAAGAGAGTTATTGGGGCTGAACAAAAAGGAAAGAAAAAAGCTACTCCTTCAACTTCAATAGCTGAGGCTGTTAATGTGATTGCGGAGACTTGCA

                1930      1940      1950      1960      1970      1980      1990      2000      2010      2020      2030      2040
          ....|....|....|....|....|....|....|....|....|....|....|....|....|....|....|....|....|....|....|....|....|....|....|....|
AC144478  AGTCGCGGAATGAGGCTATAAGTAATGCATCTATTGGTGAGGTGATGGCTGAGATTCAAACCATGGAGGCAGTTACTTCTGATTTAGAGTTTCATACAATGTGTTGTAACCTAATGATGT
          AGTCGCGGAATGAGGCTATAAGTAATGCATCTATTGGTGAAGTGATGGCTGAGATTCAAACCATGGATGCAGTTACTTCTGATTTAGAGTTTCATACAATGTGTTGTAACCTAATGATGT

                2050      2060      2070      2080      2090      2100      2110      2120      2130      2140      2150      2160
          ....|....|....|....|....|....|....|....|....|....|....|....|....|....|....|....|....|....|....|....|....|....|....|....|
AC144478  TTAAGCCAGCTAGGGAGATGTTTGTATCACTGCGGGGTTTTGAGGAAAGAAGGTTGATTTGGCTCAAATTTGCATCATTCAACCCTACTCTATTCATGAGGCCGTGATTTGGAAAAAAAA
          TTAAGCCAGCTAGGGAGATGTTTGTATCATTG

                2170      2180      2190      2200      2210      2220      2230      2240      2250      2260      2270      2280
          ....|....|....|....|....|....|....|....|....|....|....|....|....|....|....|....|....|....|....|....|....|....|....|....|
AC144478  TTGGCTCAAGAATTGGCTTAGTGATGCGTCTTGGACTTAGCTTATGCTTTTGGACTTAGTTTATGTATTTGGTTTCTTAGTTTATGTATTTGGTTGCTTAGTTTGTGTTTAGATCTTTAT

                2290      2300      2310      2320      2330      2340      2350      2360      2370      2380      2390      2400
          ....|....|....|....|....|....|....|....|....|....|....|....|....|....|....|....|....|....|....|....|....|....|....|....|
AC144478  TATATCGCACTATGTGTGAGGATTTTCCTTTATAAATTCCGTATTAAGAACTCAAGTAGTATGGTGATATAAAACCTATGTATTATGCCTTGTGAATTTGAATGAAGTGAATTTGTTACA

                2410      2420      2430      2440      2450      2460      2470      2480      2490      2500      2510      2520
          ....|....|....|....|....|....|....|....|....|....|....|....|....|....|....|....|....|....|....|....|....|....|....|....|
AC144478  TGGTTGCCTCTGTTATTTTTTATATGTCTTATGACTTATTGGTTATATTATTAAATGGTAATGGAAACTATTGTGTCTGGCAGGCAAGTGTGTACTAGTTTGTGCTAAGCTGCAAAAGGA

                2530      2540      2550      2560      2570      2580      2590      2600      2610      2620      2630      2640
          ....|....|....|....|....|....|....|....|....|....|....|....|....|....|....|....|....|....|....|....|....|....|....|....|
AC144478  TGTACTTTATGTAGATGTGTTGAAGATTGAGACTACAACAAAATTATCATCTGTGTCAGGTAATAAATTCTATGTATCTGTTATGCCTAAAAGTTCCATAGTTTAAATTGTTGATTCTGC

                2650      2660      2670      2680      2690      2700      2710      2720      2730      2740      2750      2760
          ....|....|....|....|....|....|....|....|....|....|....|....|....|....|....|....|....|....|....|....|....|....|....|....|
AC144478  TTGGAATTAGTTGGTACCTATGAATCATATCCTAATTTACATAGTGATTGACCTTTAGACATCTTATGCCTCATTTCTTTTAAGAATGTTGAGTGCATGTTTGCTTCCACGTTGCCGTGG

                2770      2780      2790      2800      2810      2820      2830      2840      2850      2860      2870      2880
          ....|....|....|....|....|....|....|....|....|....|....|....|....|....|....|....|....|....|....|....|....|....|....|....|
AC144478  AGTTGAATGTGCGGTTGGTAAACTCTACAAGACATAGCATTTATCCAGTTTTACAGTATACCAAACACACTTTGAGTTCGTCGAAGCTCAGTGAGAAACAAAACCAGTAGTAATGTATCA
```

```
                2890      2900      2910      2920      2930      2940      2950      2960      2970      2980      2990      3000
          ....|....|....|....|....|....|....|....|....|....|....|....|....|....|....|....|....|....|....|....|....|....|....|....|
AC144478  TATGGATTTTTGACGATATCACTTTTATGCCCTTGCTAGAAGTTTGAAGATTCCTTTGAATTGTTAAACCAGAATCATAACATTCTGTTCAGCTTTGCTAGTGCTATAATTAATTCATAT

                3010      3020      3030      3040      3050      3060      3070      3080      3090      3100      3110      3120
          ....|....|....|....|....|....|....|....|....|....|....|....|....|....|....|....|....|....|....|....|....|....|....|....|
AC144478  TTTGATGTTTAAAACACAGTGTAGTGCCAATGGTACTTTATTCTAGAATTTCATTTTACCTGTAGTCTAATAACATGATTATGTAAAGTAGTGGATTTTTGTTAGAGTTGCTGCAAACAT

                3130      3140      3150      3160      3170      3180      3190      3200      3210      3220      3230      3240
          ....|....|....|....|....|....|....|....|....|....|....|....|....|....|....|....|....|....|....|....|....|....|....|....|
AC144478  TGTGCTGTAAGTGTGAAGATACATGCTTATTTGAAACATATACATCACTAACCAAATTACAAACAAGATAGTTATGGGCTCAAGGGTATACAATGCACATGTATTTTGGTGCATGTATTA

                3250      3260      3270      3280      3290      3300      3310      3320      3330      3340      3350      3360
          ....|....|....|....|....|....|....|....|....|....|....|....|....|....|....|....|....|....|....|....|....|....|....|....|
AC144478  TACTGACAGATGGGATACAATGGATGGTAGTGTTTATATATTGTGATTTATTTGCTTTACTTCACAATGGATGGTGTGTTTATATATTGTCATTTACACACATGTCCACTTTATTAAAAT

                3370      3380      3390      3400      3410      3420      3430      3440      3450      3460      3470      3480
          ....|....|....|....|....|....|....|....|....|....|....|....|....|....|....|....|....|....|....|....|....|....|....|....|
AC144478  GAACTTTTGGAAGGAACAATTGGAACACAATACACAAGAAGAAGAAGATGATGATACATTTGAAGAATCCTATATTTTGGCTGCACTACTTGGTGAGTATGCAACAAAATATTTATGCAA

                3490      3500      3510      3520      3530      3540      3550      3560      3570      3580      3590      3600
          ....|....|....|....|....|....|....|....|....|....|....|....|....|....|....|....|....|....|....|....|....|....|....|....|
AC144478  AGAGCCATGTAGAACTAGTGAGCTCACAGGTCATGCATGGGTTCAAGAAATATTGCAAGGGAATCCCACTCGTTGTTATGAGATGTTTCGAATGGAAAAACATATTTTTCATAAACTTTG
                                                                       AW686181 ->     ATGAGATGTTTCGAATGGAAAAACATATTTTTCATAAACTTTG

                3610      3620      3630      3640      3650      3660      3670      3680      3690      3700      3710      3720
          ....|....|....|....|....|....|....|....|....|....|....|....|....|....|....|....|....|....|....|....|....|....|....|....|
AC144478  CCATGAATTGGTGGAACATGATTTAAAGTCTTCTAAACATATGGGGGTTGAAGAAATGGTTGCAATGTTTTTGGTCGTTGTAGGCCACGGTGTCGGTAATAGAATGATTCAAGAAAGATT
          CCATGAATTGGTGGAACATGATTTAAAGTCTTCTAAACATATGGGGGTTGAAGAAATGGTTGCAATGTTTTTGGTCGTTGTAGGCCACGGTGTCGGTAATAGAATGATTCAAGAAAGATT

                3730      3740      3750      3760      3770      3780      3790      3800      3810      3820      3830      3840
          ....|....|....|....|....|....|....|....|....|....|....|....|....|....|....|....|....|....|....|....|....|....|....|....|
AC144478  TCAACATTCGGGTGAGACTGTAAGTAGACATTTTCATCGTGTACTTCATGCATGCCTTAAGTTGTCCTTCAAATATATTAAACCCGAAGATCCTATGTTTTGTGAATGTCATGCCAAAAT
          TCAACATTCGGGTGAGACTGTAAGTAGACATTTTTATCGTGTACTTCATGCATGCCTTAAGTTGTCCTTCAAATATATTAAACCCGAAGATCCTATGTTTCGTGAATGTCATGCCAAAAT

                3850      3860      3870      3880      3890      3900      3910      3920      3930      3940      3950      3960
          ....|....|....|....|....|....|....|....|....|....|....|....|....|....|....|....|....|....|....|....|....|....|....|....|
AC144478  TAAAAATGATCAACGTTATTGGCCTTTTTTTAAGAATGCTATAGGAGTAATTGATGGTACACATGTGTCATGTGTAGTTAGTGCTAGTGAGCAACCAAGGTTTATTGGAAGAAAAGGATA
          TAAAAATGATCAACGTTATTG

                3970      3980      3990      4000      4010      4020      4030      4040      4050      4060      4070      4080
          ....|....|....|....|....|....|....|....|....|....|....|....|....|....|....|....|....|....|....|....|....|....|....|....|
AC144478  TCCAACACAAAATATTATGGCTGTATGTGATTGGAATATGTGTTTCACTTTTGTATTAGCTGGTTGGGAAGGCACTGCCCATGATGCCCGTGTTTTTGACAAAGCTCTTACTACTGCTAA

                4090      4100      4110      4120      4130      4140      4150      4160      4170      4180      4190      4200
          ....|....|....|....|....|....|....|....|....|....|....|....|....|....|....|....|....|....|....|....|....|....|....|....|
AC144478  CCTTAACTTTCCGCATCCTCCTCAAGGTATGTTATACATTGCCTTACTATATATGATTAATTTGTTATATCAATAATTAATTTATTTTCATATTTTAATAATTAATTTATTTTTAGGTAA

                4210      4220      4230      4240      4250      4260      4270      4280      4290      4300      4310      4320
          ....|....|....|....|....|....|....|....|....|....|....|....|....|....|....|....|....|....|....|....|....|....|....|....|
AC144478  GTATTATTTGGTAGATTCTGGTTATCCAACACCAATAGGGTACATTGGTCCATATAGATGTGAACGTTATCATCTTCCTGAATTTAGACGTTCAAGTGGGTTCGAAAATCATAATGAAGT

                4330      4340      4350      4360      4370      4380      4390      4400      4410      4420      4430      4440
          ....|....|....|....|....|....|....|....|....|....|....|....|....|....|....|....|....|....|....|....|....|....|....|....|
AC144478  ATTCAATTACTATCACTCAAGTTTAAGATGCACAATTGAAAGAACTTTTGGGGTATGGAAGAATAGATTTGCAATTCTACGTAGCATGCCTAAGTTCAAATATGAGACACAAGTTCATAT

                4450      4460      4470      4480      4490      4500      4510      4520      4530      4540      4550      4560
          ....|....|....|....|....|....|....|....|....|....|....|....|....|....|....|....|....|....|....|....|....|....|....|....|
AC144478  AGTTGTCGCAACAATGGCAATACACAACTTTATTAGAAGGAGTGCTGAAATGGATGTTGATTTTAATCTTTATGAAGATGAAAATACAGTCATTCACCATGATGATGATCATAGATCAAC

                4570      4580      4590      4600      4610      4620      4630      4640      4650      4660      4670      4680
          ....|....|....|....|....|....|....|....|....|....|....|....|....|....|....|....|....|....|....|....|....|....|....|....|
AC144478  TAACTTGAATCAATCCCAAAGTTTTAATGTAGCTTCTTCTTCAGAGATGGATCATGCTCGAAACTCAATCCGCGATCAAATTATAGCGTATAAGCTAAATAATTAAAAATGTAATAATTT

                4690      4700      4710      4720      4730      4740      4750      4760      4770      4780      4790      4800
          ....|....|....|....|....|....|....|....|....|....|....|....|....|....|....|....|....|....|....|....|....|....|....|....|
AC144478  TCAATATTATATTGCACTTGAACTTATATTATCTATTATCTATCTATTTGTAATATATTTTAGTTTACAATTGTGTAACTTTCTCATTTGTTTTACATCTTTTTATGTTTATATTATTTT

                4810      4820      4830      4840      4850      4860      4870      4880      4890      4900      4910      4920
          ....|....|....|....|....|....|....|....|....|....|....|....|....|....|....|....|....|....|....|....|....|....|....|....|
AC144478  ATTAAAAATCACCCAATTGATAAAAAATTATATTAGAAATAACATAAATTAATATTTTTTTAAAATTAATTTTGGAACCAATAAAATTCATGTATGATAAATTGAAATAATTTTTTAATG

                4930      4940      4950      4960      4970      4980      4990      5000      5010      5020      5030      5040
          ....|....|....|....|....|....|....|....|....|....|....|....|....|....|....|....|....|....|....|....|....|....|....|....|
AC144478  AGAATGAGTTTTAAATACAATTAAAAGGATAGTTTAGTCATTACACATTCAAAATCAATTTTGATTCAAACTATCCAAACAACATCAACTCACATGAATCACTTTTAATAAAGTGTATCC

                5050      5060      5070      5080      5090      5100      5110      5120      5130      5140
          ....|....|....|....|....|....|....|....|....|....|....|....|....|....|....|....|....|....|....|....|....|.
AC144478  AAACATAATCAATTCACATTCAACTCACTTTTAACCAAAATCAATTCTCTCAAAATTAATTCTATCAAAATCAATTCTCCCCGCCGCCATACCAAACACACACTTA
```
